# Dupilumab‐Related Adverse Events and Intolerance in Aspirin‐Exacerbated Respiratory Disease Patients

**DOI:** 10.1002/alr.70129

**Published:** 2026-03-07

**Authors:** Lancelot P. Herpin, Randy Bach, Ricardo Bruneto, Kerol A. Faltas, Michael A. Kohanski, James N. Palmer, Nithin D. Adappa, Jennifer E. Douglas, John V. Bosso

**Affiliations:** ^1^ Perelman School of Medicine University of Pennsylvania Philadelphia Pennsylvania USA; ^2^ Division of Rhinology Department of Otorhinolaryngology‐Head and Neck Surgery University of Pennsylvania, Perelman School of Medicine Philadelphia Pennsylvania USA; ^3^ Department of Ophthalmology, Otolaryngology, and Head and Neck Surgery University of São Paulo, Ribeirão Preto Medical School São Paulo Brazil

**Keywords:** adverse event, aspirin‐exacerbated respiratory disease, autoimmune reaction, dupilumab

## 1 Introduction

Aspirin‐exacerbated respiratory disease (AERD), also known as Samter's triad, is a chronic inflammatory condition characterized by asthma, chronic rhinosinusitis with nasal polyposis (CRSwNP), and respiratory reactions to cyclooxygenase‐1 inhibitors. Patients with AERD frequently experience severe, treatment‐refractory disease marked by rapid nasal polyp recurrence, frequent asthma exacerbations, impaired olfaction, and reduced quality of life despite multimodal medical and surgical management [1]. Disease severity and recurrence following endoscopic sinus surgery contribute to substantial healthcare utilization in this population [1].

**Key Points**

- Dupilumab intolerance occurred in 17% of aspirin‐exacerbated respiratory disease patients in practice, higher than prior reports.
- Joint pain and hypereosinophilia were the most common causes of treatment discontinuation.
- Rare but severe autoimmune reactions occurred, underscoring the need for early monitoring.

Aspirin therapy after desensitization (ATAD) represents an established disease‐modifying treatment option for AERD, though long‐term tolerance and adherence vary across patients [2]. The emergence of biologic therapies targeting type 2 inflammation has expanded treatment options for patients with CRSwNP and asthma [3]. Dupilumab, a monoclonal antibody targeting the IL‐4 receptor alpha subunit to inhibit IL‐4 and IL‐13 signaling, has demonstrated efficacy in reducing nasal polyp burden and improving asthma control in patients with chronic rhinosinusitis with nasal polyposis, including those with AERD [3, 4].

However, patients with AERD remain underrepresented in dupilumab clinical trials, limiting the generalizability of safety data to this subgroup [4, 5]. Emerging reports suggest that dupilumab may be associated with distinct adverse events (AEs), including hypereosinophilia, musculoskeletal symptoms, dermatologic reactions, and autoimmune phenomena [5, 6]. When selecting between treatment options such as aspiration therapy after desensitization and biologic therapy, both treatment efficacy and tolerance are central considerations. Recent data report an overall ATAD tolerance rate of 82.7%, with tolerance varying by sex, menopausal status, and race [7]. To meaningfully compare these therapeutic pathways and identify which patients may benefit most from each, it is essential to define dupilumab‐related adverse events, dupilumab tolerability, and discontinuation rates in real‐world AERD cohorts [2]. This study aims to quantify and characterize dupilumab‐related AEs in AERD patients treated at a tertiary care center, providing insight into its real‐world safety profile and informing individualized treatment decisions.

## 2 Methods

This retrospective chart review was conducted at the University of Pennsylvania and was determined to be Institutional Review Board‐exempt. Adult patients with a clinical diagnosis of AERD, defined according to international consensus criteria, who received at least one dose of dupilumab between November 2018 and June 2025 were included [8]. Diagnosis was confirmed using ICD‐10 coding and allergist documentation. Patients were excluded if post‐treatment follow‐up data were unavailable.

Collected variables included age, sex, race and ethnicity, autoimmune disease history, dupilumab treatment duration, documented AEs, and reasons for treatment discontinuation. AEs were attributed to dupilumab based on treating physician documentation in the medical record.

Statistical analyses were performed using R version 4.0.3. Continuous variables were assessed for normality and compared using ANOVA or Kruskal–Wallis testing as appropriate. Categorical variables were analyzed using Fisher exact testing. A two‐tailed hypothesis was adopted, and a *p*‐value ≤0.05 was considered statistically significant. When overall tests were significant, post hoc analyses were performed using Bonferroni correction.

## 3 Results

Among 104 patients with AERD treated with dupilumab, 26 (25.0%) experienced at least one dupilumab‐related AE. Eighteen patients (17.3% of the total cohort; 69.2% of those with AEs) discontinued dupilumab because of an AE (Table 1). Four additional patients discontinued therapy for non‐AE reasons, including cost, pregnancy, and lack of effectiveness.

**TABLE 1:** Clinical and demographic information for AERD patients.

Patient	No adverse event	AE without discontinuation	AE with discontinuation	*p*‐value
Patients, n	78	8	18	
Age, mean (SD), years	50.6 (12.7)	50.8 (10.1)	50.4 (12.2)	0.995
Sex, *n* (%)				
Female	47 (60.3)	7 (87.5)	14 (77.8)	0.176
Male	31 (39.7)	1 (12.5)	4 (22.2)	
Race, *n* (%)				
Caucasian	44 (56.4)	7 (87.5)	13 (72.2)	
African‐American	19 (24.4)	0 (0.0)	2 (11.1)	
Asian	5 (6.4)	1 (12.5)	0 (0.0)	0.101
Hispanic/Latino	1 (1.3)	0 (0.0)	2 (11.1)	
Unknown/Other	9 (11.5)	0 (0.0)	1 (5.6)	
Autoimmune conditions, *n* (%)				
No	65 (83.3)	8 (100.0)	12 (66.7)	0.129
Yes	13 (16.7)	0 (0.0)	6 (33.3)	
Dupilumab duration, mean (SD), months	37.5 (20.3)	41.1 (23.0)	11.5 (10.0)	<0.0001
Adverse event				
Joint pain		0 (0.0)	9 (50.0)	
Hypereosinophilia		0 (0.0)	4 (22.2)	
Injection site rash		1 (12.5)	4 (22.2)	
Body rash		2 (15.0)	3 (16.7)	
Vision issues		5 (62.5)	3 (16.7)	
Cardiac issues		0 (0.0)	2 (11.1)	
Myalgia		0 (0.0)	2 (11.1)	
More infections		2 (25.0)	0 (0.0)	
Urticaria		1 (12.5)	1 (5.6)	
Lupus‐like reaction		0 (0.0)	1 (5.6)	
Hair loss		0 (0.0)	1 (5.6)	
Constitutional symptoms		0 (0.0)	1 (5.6)	
Skin sensitivity		0 (0.0)	1 (5.6)	
Sweet's syndrome		0 (0.0)	1 (5.6)	

*Note*: Information for AERD patients with no adverse event, at least one adverse event without discontinuation, and at least one adverse event resulting in discontinuation, and frequency of AEs without and with discontinuation.

Abbreviations: AE, adverse event; AERD, aspirin‐exacerbated respiratory disease.

After adverse event‐related discontinuation, 12 patients switched to another biologic therapy, most commonly mepolizumab or tezepelumab, while one patient underwent aspirin desensitization (Table 2). The remaining patients did not pursue additional biologic therapy.

**TABLE 2:** Clinical case summaries of AERD patients with AEs.

Age	Sex	Race	AI	Time to 1st AE^a^	Dupilumab duration^a^	AE	Treatment	Biologic switch to
**Patients who experienced AEs with discontinuation**								
65.1	M	Caucasian	No	13.7	17.6	Joint pain, myalgia	Stop drug	None
51.8	F	Caucasian	No	0	15.6	Severe joint and hip pain, urticaria	Reduce dose, Stop drug	Tezepelumab
40.3	F	Hispanic/Latino	No	3.4	4.9	Generalized joint pain, bilateral foot pain	Stop drug	Mepolizumab
33.8	M	Caucasian	No	17.8	27.2	Chills, hyperhydrosis	Stop drug	Tezepelumab
52.4	F	Unreported	Hypothyroidism	NA	20.6	Bright lights, visual aura, rash	Stop drug	Mepolizumab
62.8	F	Caucasian	Sjogren syndrome	NA	1.4	Severe joint pain, palpitations	Stop drug	None
65.5	F	Caucasian	No	11.9	17.8	Visual bluriness, tender injection‐site rash	Stop drug	None
66.3	F	African American	No	0	0.5	Allergic injection‐site rash	Prednisone, stop drug	None
49.2	F	Hispanic/Latino	No	NA	14	Skin sensitivity	Stop drug	None
56.2	F	Caucasian	Hypothyroidism, psoriatic arthritis, juvenile type 1 diabetes	0.5	0.46	Hypersensitiivty injection‐site rash, visual wavy lines, and increased bluriness	Zyrtec/Steroid cream, stop drug	Benralizumab, Tezepelumab
60	M	Caucasian	No	6.8	6.7	Severe eosinophilia	Stop drug	Mepolizumab
35.1	M	Caucasian	No	12.2	35.4	Joint pain	Stop drug	Mepolizumab, ATAD
48.3	F	Caucasian	Sjogren syndrome	5.2	5.1	Eosinophils >1500	Stop drug	None
33.2	F	Caucasian	No	14	14.4	Allergic tender rash	Zyrtec/Benadryl, stop drug	Mepolizumab
47.3	F	African American	No	NA	5.3	Allergic injection‐site rash, severe joint pain	Stop drug	Mepolizumab
59.7	F	Caucasian	No	0.5	16.5	Drug‐induced Lupus‐like syndrome, severe joint pain, hair loss	Reduce dose, stop drug	Mepolizumab
27	F	Caucasian	EGPA	0.9	3	Eosinophilia, joint pain	Steroids, stop drug	Mepolizumab
52.6	F	Caucasian	IgA nephropathy	0.9	1.3	Eosinophils >13,000, joint pain, myalgia, Sweet syndrome, developed eosinophilic myocarditis (hospitalized)	Steroids, stop drug	Mepolizumab
**Patients who experience AEs without discontinuation**								
39.8	F	Caucasian	No	11.8	34.4	More infections, local rash, rosacea, vision issues	Metronidazole and then ketoconazole	
61.7	F	Caucasian	No	37	55.3	Increased infections	None	
60	M	Caucasian	No	0	70.7	Urticaria	None	
47.7	F	Caucasian	No	NA	37.5	Increased visual bluriness	None	
38.8	F	Asian	No	NA	18.6	Right eye pruritus and epiphora	Eye drops	
63.9	F	Caucasian	No	36.8	48	Dry eyes	Reduce dose	
42.5	F	Caucasian	No	NA	62.7	Very mild dry eye	None	
52.2	F	Caucasian	No	NA	1.7	Rosacea	Metronidazole	

Abbreviations: AE, adverse event; AERD, aspirin‐exacerbated respiratory disease; AI, autoimmune conditions; ATAD, aspirin therapy after desensitization.

^a^ Time in months.

The most common AEs leading to discontinuation were joint pain, hypereosinophilia, dermatologic reactions, and vision changes (Table 1). Rare severe AEs included drug‐induced systemic lupus erythematosus and Sweet's syndrome with eosinophilic myocarditis (Table 2).

Clinical and demographic characteristics by AE status are shown in Table 1. Age and sex did not differ significantly between groups. Autoimmune disease history and race demonstrated trends toward association with AE‐related discontinuation without reaching statistical significance.

## 4 Discussion

Adverse event‐related dupilumab discontinuation has not been adequately characterized in AERD, an important gap given underrepresentation in CRSwNP trials, heightened baseline type 2 inflammation compared to CRSwNP, and the availability of ATAD as an alternative, making tolerability central to individualized treatment selection [4, 5]. In this real‐world AERD cohort, 17.3% discontinued dupilumab due to AEs, exceeding rates reported in Phase III clinical trials and real‐world studies of broader CRSwNP populations [3, 9].

The most common AEs prompting discontinuation in this cohort were musculoskeletal symptoms, hypereosinophilia, dermatologic reactions, and vision changes, consistent with prior real‐world and pharmacovigilance reports [5, 6]. Rare but severe autoimmune complications were observed. One possible mechanism is immune dysregulation following IL‐4 and IL‐13 blockade, resulting in Th17‐skewed inflammatory responses and eosinophil‐mediated tissue injury. Bridgewood et al. reported that IL‐4/IL‐13 antagonism was associated with Th17‐skewed immune responses, manifesting as increased inflammatory skin and joint disease, as well as IL‐5‐mediated eosinophilic disorders [10]. The higher rate of AE‐related dupilumab discontinuation in our AERD cohort may reflect a higher baseline type 2 inflammatory burden than CRSwNP, possibly leading to more pronounced inflammatory shifts after IL‐4/IL‐13 blockade and a higher likelihood of clinically significant adverse events requiring discontinuation.

This study is limited by its retrospective design, single‐center setting, and modest sample size, which may limit the power to detect predictors of intolerance. Higher AEs and discontinuation rates may reflect ascertainment bias via closer tertiary center monitoring and easier access to alternative therapies. Nonetheless, it provides clinically meaningful insight into dupilumab tolerability in AERD and underscores the need for larger, multi‐institutional studies to better define risk factors and mechanisms of AEs.

## Funding

This research was supported by the Thomas B. McCabe and Jeannette E. Laws McCabe Fund at the University of Pennsylvania.

## Conflicts of Interest

The authors declare no conflicts of interest.
